# The Utility of Multimodal Imaging and Artificial Intelligence Algorithms for Overlying Two Volumes in the Decision Chain for the Treatment of Complex Pathologies in Interventional Neuroradiology—A Case Series Study

**DOI:** 10.3390/life13030784

**Published:** 2023-03-14

**Authors:** Bogdan Valeriu Popa, Aurelian Costin Minoiu, Catalin Juratu, Cristina Fulgoi, Dragos Trifan, Adrian Tutelca, Dana Crisinescu, Dan Adrian Popica, Cristian Mihalea, Horia Ples

**Affiliations:** 1 Department of Radiology, Clinical Emergency Hospital Bucharest, 014461 Bucharest, Romania; 2 Department of Radiology, Carol Davila University of Medicine and Pharmacy, 014461 Bucharest, Romania; 3 Department of Radiology, “Pius Brinzeu” County Emergency Clinical Hospital, 300723 Timisoara, Romania; 4 Department of Interventional Neuroradiology—NEURI Brain Vascular Center, Bicêtre Hospital, Assistance Publique Hopitaux de Paris, 94270 Paris, France; 5 Department of Neurosurgery, University of Medicine and Pharmacy “Victor Babes”, 300041 Timisoara, Romania; 6 Department of Neurosurgery, “Pius Brinzeu” County Emergency Clinical Hospital, 300723 Timisoara, Romania

**Keywords:** interventional neuroradiology, automatic overlay, 3D rotational angiography, aneurysm, endovascular treatment

## Abstract

3D rotational angiography is now increasingly used in routine neuroendovascular procedures––in particular, for situations where the analysis of two overlayed sets of volume imaging proves useful for planning the treatment strategy or for confirming the optimal apposition of the intravascular devices used. The aim of this study is to identify and describe the decision algorithm for which the overlay function of 3D rotational angiography volumes, high-resolution contrast-enhanced flat panel detector CT adapted for intravascular devices (VasoCT/DynaCT), non-enhanced flat detector C-arm volume acquisition functionality integrated with the angiography equipment (XperCT/DynaCT), and isovolumetric MRI volumes were all used in treatments performed in a series of 29 patients. Two superposed 3DRA volumes were used in the treatment aneurysms located at the junction of two vascular territories and for arteriovenous malformations with compartments fed from different vascular territories. The superposition function of a preoperatively acquired 3DRA volume and a postoperatively acquired VasoCT volume provides accurate information about the apposition of neuroendovascular endoprostheses used in the treatment of aneurysms. The automatic overlay function generated by the 3D workstation is particularly useful, but in about 50% of cases it requires manual operator-dependent correction, requiring a certain level of experience. In our experience, multimodal imaging brings an important benefit, both in the treatment decision algorithm and in the assessment of neuroendovascular treatment efficacy.

## 1. Introduction

In the last decade, neuroendovascular treatments using novel techniques (FD stent, intrasaccular devices, stent-assisted coiling) have seen an increase in the overall share of cerebral aneurysm treatments, leading to the need for augmentation of complex imaging analysis both pre- and post-treatment to validate the efficacy of these types of treatment.

Cerebrovascular anomalies, such as saccular aneurysms or arteriovenous malformations, are life-threatening pathologies and often asymptomatic, and the rupture of which represents a devastating event with increased risk of morbidity and mortality. Medical imaging and especially interventional neuroradiology play a central role in much of the medical pathway of a patient with such a condition, from diagnosis to treatment as well as follow up.

Artificial intelligence (AI), through its subfields machine learning (ML), and deep learning (DL), through their supra-human capability to process vast datasets and to be trained using them, both show great promise in supporting the medical journey of patients affected by cerebrovascular malformations. Studies so far have shown how artificial intelligence can be helpful in predicting the risk of acquiring such conditions, detecting them, predicting the risk of rupture, predicting post-rupture complications, selecting the therapeutic strategy, or predicting the therapeutic outcome, with a huge potential to improve the patient’s outcome [1].

Experimental studies with artificial intelligence systems have demonstrated their efficacy and safety in the diagnosis and treatment of cerebrovascular malformations [2]. Pre-procedural simulations also increase the average performance of the interventionist physician. Established advantages of artificial intelligence systems include improved catheter tip stability, which will decrease the number of required movements and thus operating times and risk of complications, ease of navigation through tortuous vascular anatomy, and decrease radiation exposure [3]. 3D angiography based on artificial intelligence is a reliable method of anatomical analysis of cerebrovascular anatomy that can further reduce patient irradiation [4]. More advanced algorithms may also allow road-mapping of the cerebrovascular vasculature without contrast media. Real-time AI algorithms can superimpose high-resolution pre-procedural images (CT, MRI) with per-procedural fluoroscopy images, thus serving as a true guide to the interventionist during catheter manipulation [5]. Despite the exponential evolution of the field of artificial intelligence and its vast applicability in medicine, and especially in imaging, the technology is still in its infancy. Thus, before it can be routinely used in clinical settings, it needs to be thoroughly tested and validated.

In case the aneurysmal neck is located at the confluence of two different vascular territories (anterior communicating artery with both A1 segments patent and vertebrobasilar junction aneurysms involving both vertebral arteries), the simple analysis of 2D angiographic images is not sufficient for understanding the regional anatomy or the post-treatment apposition of devices that are placed at the border between two vascular territories.

In the pathology of cerebral AVMs whose nidus is fed by different vascular territories (frontal AVMs fed by both ACAs, temporoparietal AVMs fed by ICA and VA or posterior fossa AVMs), the analysis of the 3DRA overlapping volumes of these vascular territories provides detailed information on the compartmentalization of the nidus. Thus, a thorough analysis of this type of imaging gives the possibility to consider a specific compartment over the second one in case of multiple session embolizations. The current attitude for hybrid treatment (neurosurgical-neuroendovascular) is to favour the deep compartments, which are difficult to access with neurosurgical therapy. In case of the detection of intranidal aneurysms, the identification of the feeding artery irrigating this compartment is important for prioritizing endovascular treatment.

Post-endovascular treatment analysis involves superimposing a pre-treatment 3DRA volume with a VasoCT or XperCT volume acquired post-operatively. Regarding WEB or contour intrasacular devices, the analysis of the pre- and post-operative imaging overlay provides certainty regarding the proper placement, and it quantifies the degree of protrusion of the device into the parental vessel of the aneurysm.

The 3D superimposition with the MRI volume in case of AVMs gives an overview of the relationship between the circulated nidus, the porencephalic cavity in case of ruptured AVMs, and the neuraxial structures adjacent to the nidus. It also helps to understand the anatomy during patient follow up by accurately identifying the increase in volume of the malformation or by identifying the occurrence of risk factors, such as intranidal aneurysms.

An important consideration in the case of both pre- and post-operative volume overlay is that, by its nature, a stent can alter the regional anatomy by straightening the vessel, so the automatic overlay function can provide an erroneous image, which is corrigible by the operator’s use of manual repositioning, especially based on aneursymal sac, and, in this way, the regional anatomy and especially the neck is corelated between the two volumes.

Due to the fact that the automatic software is using bone landmarks, it will not adapt to the vascular anatomy changes caused by the endovascular device (especially with stents). In this situation, to avoid a misleading conclusion, we have to manually adjust the volumes by using the aneurysmal sac and the parent vessel as landmarks.

## 2. Materials and Methods

We retrospectively enrolled 29 patients from a database of 170 interventions performed between 2017 and 2022 in which these imaging analysis methods were used. In these 29 patients, simple analysis of 2D angiographic images could not provide the anatomical details desired by the neurointerventional radiology team.

Analysis of images obtained by superimposing two 3DRA volumes was performed in 6 patients with cerebral aneurysm and 6 patients with AVMs and DAVFs.

Biplanar angiography was performed standardly with Philips Allura FD 20 and Azurion FD 20 monoplane angiograph.

### 2.1. 3D Rotational Angiography

The 3D Rotational Angiography (3DRA) examination was optimized for high-resolution reconstructions (either volume-rendering or multiplanar mode) and also for the visualization of iodine contrast, as 3D rotational angiography is meant for larger contrast differences (with pure contrast medium). The tube voltage was fixed at approximately 80 kV and a total volume between 24 to 28 mL of iodine contrast material (Visipaque 270; GE Healthcare, Mississauga, ON, Canada), at a rate of 4 mL/s, was injected. The rotation started 1.5 s after the injection in the internal carotid artery or vertebral artery [6]. The 3D reconstruction, 120 two-dimensional (2D) images were registered at a rate of 30 frames/s, from −120° to +120°, at a speed of 55°/s, with an acquisition time of 4 s and a reconstruction time of 5 s. The datasets acquired into a 2563 pixel matrix were directly transferred to a 3D workstation (Xtravision, Philips Healthcare, Amsterdam, The Netherlands), and reconstruction of the 3D volume was made from 100% to 140% in order to obtain more information with a better resolution [7].

### 2.2. VasoCT

Similar to 3DRA, VasoCT uses approximately 80 kV for the X-ray tube and is also optimized for high-resolution reconstructions and for the visualization of iodine contrast, especially for small contrast differences resulting in diluted contrast medium. In order to achieve high contrast resolution, VasoCT acquires a larger number of images—more precisely, 620. As a result, the acquisition takes longer—20 s—and the reconstruction time is approximately 26 s. Because of its superior contrast resolution, VasoCT is the most appropriate technique for use with endovascular devices. On the other hand, VasoCT examination performed with the assistance of the angiography system allows the reduction of metallic artifacts and also the detailed analysis of intracerebral vessels [6]. 

### 2.3. 3D Workstation

A C-arm angiography unit (Azurion/Allura Xper FD20, Philips Healthcare) connected to a dedicated 3D workstation (XtraVision, Philips Healthcare) was used. The patient was placed in a supine position in the angiography chamber. The patient’s head was fixed with the aid of a headset in the neutral position. The isocenter was set at the level of the target lesion (aneurysm, AVM nidus). This primary dataset was then automatically transmitted to the workstation; the volume reconstruction appeared on the Workstation 8 s after the acquisition was finalized. We overlaid the primary dataset (3DRA, VasoCT, XperCT) with the secondary dataset (VasoCT, XperCT, MRI, 3DRA). Either manual or automated methods can be used to merge the vascular and anatomical landmarks. Sometimes, a fully manually controlled overlay is particularly useful if the automach function fails to overlay volumes. In order to perform a controlled manual merge of two volumes accurately, a few tips and tricks are needed to make the final result useful in the decision algorithm. At the moment, only the initial software version (3DRA) of the dedicated workstation allows the merging of two imaging volumes, while the software version entitled SmartCT does not yet feature this function.

**Figure 1:**
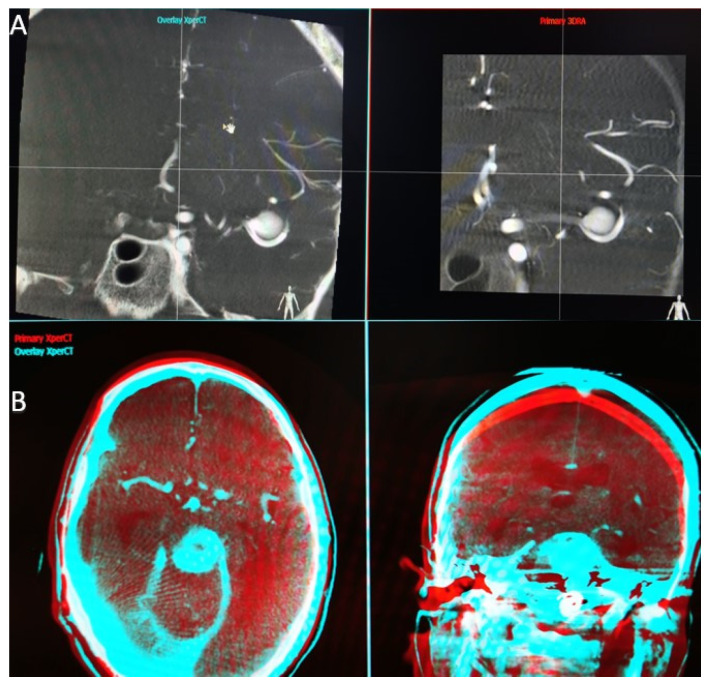
(**A**) Workstation first interface that parallels the two volumes we have selected. (**B**) Workstation second interface, red—primary volume, and blue—overlay volume, two spatial planes at the same time.

When the application that performs the overlay function is launched, we find a first interface that parallels the two volumes we have selected (Figure 1A). At this step, we have to choose a high Hounsfield unit object landmark (bone, endosaccular device, stent, coils) that we use to synchronize the volumes in all 3 planes (axial, sagittal, and coronal). Once the target point is placed in the same incidence on both volumes and in all 3 planes, we can open the second interface.

In the second interface (Figure 1B), the screen is also split into two parts, and we have the possibility to analyze the superposition of the two volumes in different colors (red—primary volume, and blue—overlay volume) and in two spatial planes at the same time. Both volume images were presented in different colors and in the same window as the orthogonal multi-planar reconstruction (MPR) images in axial, sagittal, and coronal orientations. We can now manually correct angulation errors (higher in patients without general anesthesia) and accurately calibrate the anatomy, thereby compensating for vascular anatomical changes due to stent placement.

The automatic overlay function is more efficient in cases where the spatial landmarks were selected manually in the first interface, as it avoids large mismatches that cannot be fully compensated by the automatic software.

Initially, the auto-mach function was used (the process of automatic overlaying using the artificial intelligence algorithms described above), which makes the two volumes overlay independently and takes about 30 s per case. This function has the advantage of a high accuracy, if the FOV is at least 22 cm. Within this volume, there are sufficient bone landmarks (base of skull, temporal petrous part, etc.), and the disadvantage that in distal lesions (fronto-parieto-occipital region), due to the lack of bone landmarks, the automatic registration is not acheived accurately, making manual intervention necessary.

If the patient is under general anesthesia, the auto-mach function has a high accuracy because the position of the patient’s head remains identical between the 2 acquisitions, whereas in a patient under local anesthesia there may be changes in rotation and angulation of the head between the 2 acquisitions. An important point to note is that in 3DRA volumes, both volumes must be acquired with the same FOV, as the current software does not allow overlapping in case of volumes acquired with different FOV. On the other hand, a 3DRA volume purchased with any of the available FOVs can be overlayed with a VasoCT volume that has a standard FOV of 22 or 27 cm.

### 2.4. The 3D-3D Registration Algorithm

The 3D–3D registration algorithm applied in the Interventional Workstation (Philips, Best, The Netherlands) searches the optimal rigid transformation according to a similarity measure, meaning that it translates and rotates the 3D dataset in 6 degrees of freedom to find the best match, but it does not deform the dataset. It employs Mutual Information [8] as a similarity metric, and the Powell algorithm [9] as an optimizer to find the best match. Mutual Information, in this case, expresses the mutual dependence between the grey values in both datasets for corresponding voxel locations. For the best matching spatial transformation, the corresponding anatomical structures map onto each other, and, therefore, a larger dependency between their grey values is reached. This metric is particularly useful for multi-modality registration, as it does not make any assumptions on which grey value in one dataset should match which grey value in the other.

In order to increase the capture range (the maximal distance between the datasets when starting the registration algorithm that still leads to a successful result), a multi-resolution scheme is applied [10]. First, the Powell algorithm is run with the 3DRA data downsampled to 64^3^ voxels, and the multi-modal data are downsampled to match the 3DRA voxel size in mm. Then the registration process is repeated with the 3DRA data downsampled to 128^3^ voxels and a matching voxel size for the multi-modal data. For both datasets, 256 grey value bins are used [11]. Experimental results using a realistic head phantom yielded an average registration error of 0.515 mm and an average rotation error of 0.241° [12]. The capture range was determined using clinical data. For 3DRA-CT registration, 88% of the CT datasets could be registered successfully when the registration process was started within 30 mm translation offset. A total of 67% managed to robustly register within 50 mm translation. A total of 88% of the CT datasets still could be registered correctly to the 3DRA dataset when the initial rotation offset is 20°, and 74% when the rotation offset is 30°. For 3DRA-MR registration, 80% of the MR datasets can be registered correctly with an initial translation offset of 15 mm, and 84% yield correct results when the registration is started within 20° rotation. In order to accelerate the registration algorithm, the GPU is used, leading to computation times of less than 8 s.

### 2.5. Angiography Table Position Recall

With the angiography system used, there is the possibility to place the angiography table in the same position as the previous acquisition. Using this function, we can recall the position used for the 3DRA volume performed during diagnosis to make the post-treatment VasoCT acquisition. In this way, with the patient under general anesthesia, we can obtain two volumes with almost identical spatial landmarks, and the artificial intelligence software can do an efficient automatic superposition, without the need for manual adaptation, thus saving valuable time in image analysis. This function is particularly important because, by quickly targeting the good apposition of the device, we can withdraw the catheters and the microcatheters in a safe position and thereby reduce the risk of an intraoperative complication (formation of an endoluminal thrombus).

## 3. Results

Out of the total cerebral aneurysms, five were anterior communicating artery aneurysms, a location in which understanding the anatomy of the anterior communicating complex and treatment planning were not possible by separate analysis of the two internal carotid axes.

One aneurysm was a vertebrobasilar junction aneurysm, in which the fusiform and dysplastic aspect of the aneurysm sac required thorough planning, by taking into account all of the regional anatomical aspects.

For four patients with AVM, the function was necessary for analyzing the compartments of the nidus fed from several vascular territories, and was thus essential in the choice of the vessels from which embolization would be performed, taking into consideration the safety aspects in terms of navigation and the brain territory involved.

For the two patients with DAVF fed from different vascular territories, operative planning to reach the target arterio-venous junction area was only possible after analysis of the superimposed images.

In nine patients, preoperative 3DRA volume with postoperative vasoCT superimposition was used, which is particularly useful in assessing the good apposition of FD or intrasaccular devices (WEB). 

In three patients with AVM and DAVF, 3DRA volume was superimposed with MRI isovolumetric volume or with the final procedure XperCT volume. Superposition with previous MRI imaging was necessary to understand the relationships between the nidal compartment and adjacent brain tissue. The XperCT volume at the end of the procedure was superimposed with the initial 3DRA volume to understand the location of the cast of embolic material in the AVM nidus, which is especially useful in cases where the embolization is performed in multiple sessions.

The overlaying of two XperCT volumes (pre-operative and post-operative) was used in five cases to assess the evolution of hydrocephalus in the case of ruptured aneurysms. This function also proved to be very useful for detecting intraoperative bleeding, especially small hemorrhages by comparing the initial volume of the subarachnoid hemorrhage with the volume present by the end of the operation.

Among the 29 patients enrolled, for 24 of them (83%) we used the automatic image overlay function. Out of the 24 patients with automatic overlay, in 11 (45%) patients, manual image adjustment was necessary—in 4 of them due to the lack of suitable landmarks (necessary for the automatic software to synchronize the planes) and in 7 due to changes in vascular anatomy by the implanted stents.

In all five cases (17%) where we used only the manual overlay function, AVMs were involved. Their location in areas without any bony landmarks and the use of MRI imaging resulted in the impossibility of automated software synchronizing the volumes.

### 3.1. Case 1

**Figure 2:**
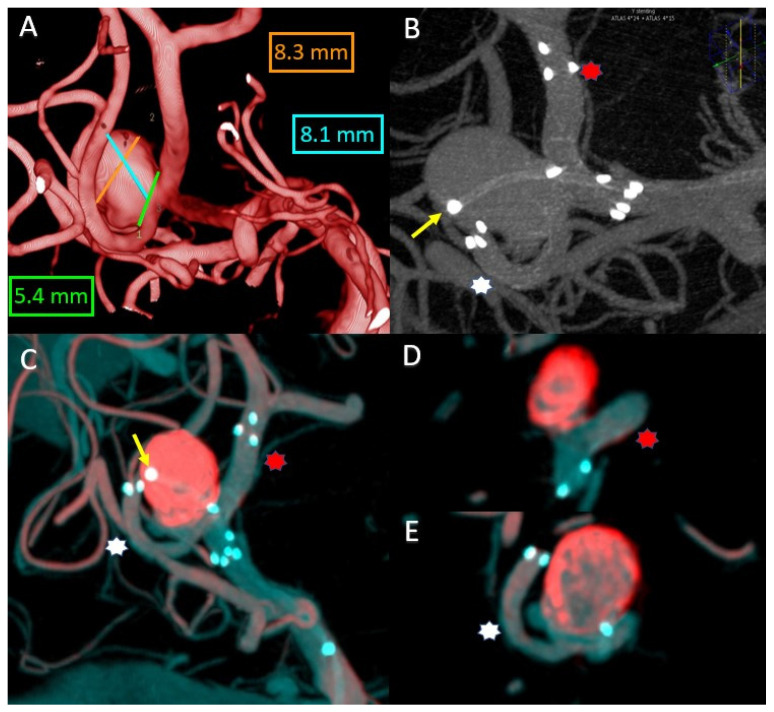
(**A**) Right MCA bifurcation aneurysm 3DRA size. (**B**) VasoCT MIP after stents deployment. (**C**) MIP Overlay between the 3DRA volume from the diagnostic phase (red color) and the VasoCT (blue color) after stents deployment. (**D**) Thin-slice image reconstructions overlay between the 3DRA volume from the diagnostic phase (red color) and the VasoCT (blue color)—aneurysmal neck protection by superior M2 branch stent. (**E**) Thin-slice image reconstructions overlay between the 3DRA volume from the diagnostic phase (red color) and the VasoCT (blue color)—aneurysmal neck protection by inferior M2 branch stent. Yellow arrow—coiling microcatheter jailed in the aneurysmal sac. Red asterisk—superior M2 branch with 4 × 24 mm laser cut stent. White asterisk—inferior M2 branch with 4 × 15 mm laser cut stent.

Here, we are talking about a 37-year-old patient treated for two cerebral aneurysms, one on the tip of the basilar artery and one on the bifurcation of the right middle cerebral artery. The subject of the presentation is the right middle cerebral artery aneurysm, which, being on a wide neck, was treated by the Y-stenting technique along with coil placement in the aneurysmal sac (Figure 2A,B). 

It is important in this case to highlight the correct positioning of the stents before the coil placement stage. To understand their positioning, we performed an overlay between the initial 3DRA volume and the VasoCT examination performed after the placement of both laser cut stents (Figure 2C). We observed that both stents are well open and protect the aneurysmal neck and, at this point, we can be sure that we can safely proceed with the intervention (Figure 2D,E).

### 3.2. Case 2

**Figure 3:**
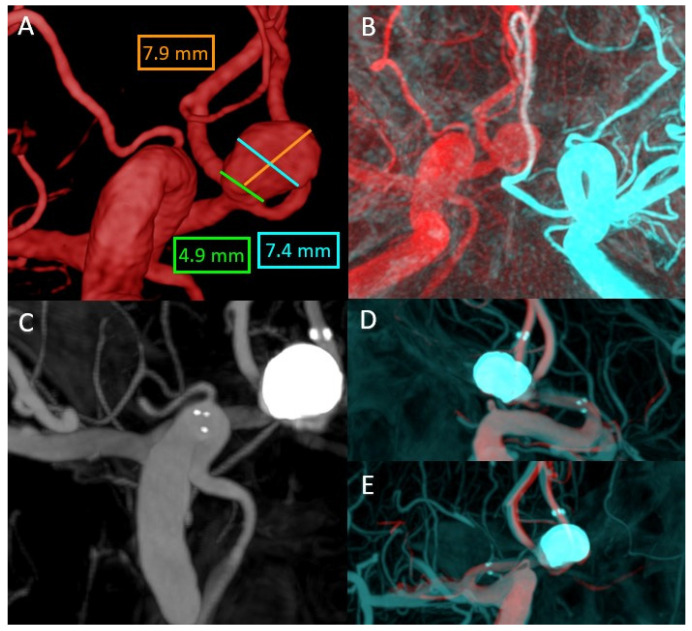
(**A**) Anterior communicating artery (ACoA) aneurysm size—right ICA 3DRA volume. (**B**) Overlay between the right ICA 3DRA volume and the left ICA 3DRA volume. (**C**) VasoCT MIP after stent deployment. (**D**,**E**) MIP Overlay between the VasoCT after the stent deployment and the right ICA 3DRA initial volume.

This case concerns a 39-year-old patient with an incidental finding of an anterior communicating aneurysm. The anatomy of the anterior communicating complex and the fact that the aneurysm had a wide neck (Figure 3A) made it necessary in this case regarding the overlying function, both in the pre-treatment planning, to overlay the two 3DRA volumes between the left and right carotid axis (Figure 3B), and then at the end of the treatment between the initial right carotid 3DRA volume and the final VasoCT volume. 

In the treatment planning stage, after analysis of the images obtained by overlay, it was decided to place a laser cut stent between the right A1 segment and the left A2 segment, thus achieving an adequate coverage of the aneurysmal collection in order to place the coils later (Figure 3C). Due to flow competition, it would also have been difficult to obtain an actual size for the left A2 segment diameter without the use of this software, which is mandatory for choosing the right stent. After the treatment, to show good apposition and patency of the stent and the vascular branches involved, it was necessary to analyze simultaneously the 3DRA and VasoCT volumes (Figure 3D,E).

### 3.3. Case 3

**Figure 4:**
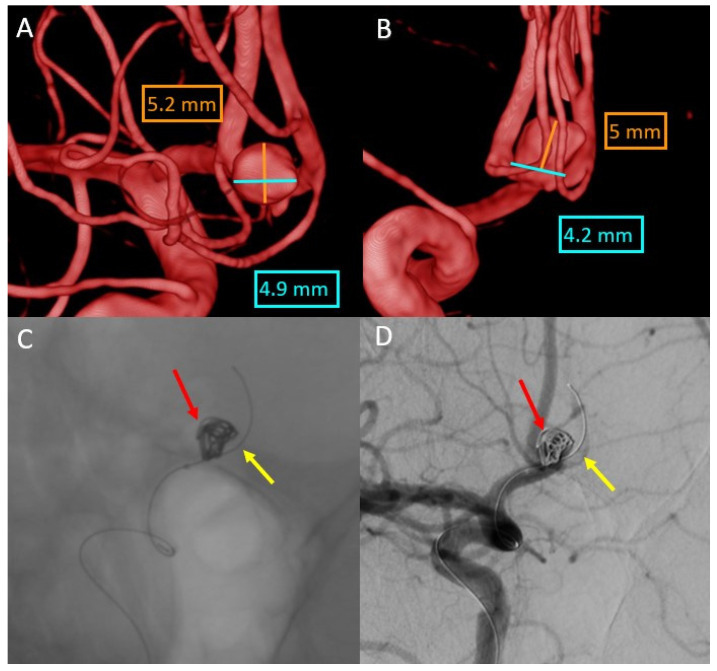
(**A**,**B**) Anterior communicating artery (ACoA) aneurysm size—right internal carotid artery 3DRA volume. (**C**,**D**) DSA images ACoA aneurysm balloon-assisted coil embolization (red arrow—coils; yellow arrow—balloon), placed in contralateral A2 segment.

We present a 48-year-old patient with a subarachnoid hemorrhage Fisher IV through a rupture of the anterior communicating artery aneurysm. The aneurysm with a diameter of 4.9 × 5.2 mm, a height of 5 mm, and a neck of 4.2 mm was treated with a balloon-assisted coil embolization (Figure 4).

**Figure 5:**
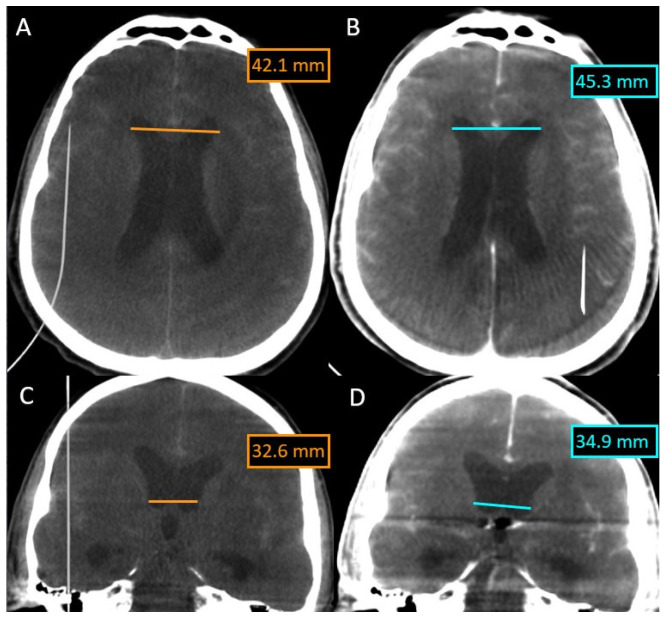
Evolution of hydrocephalus—comparison with the Overlay software. (**A**) XperCT volume axial preoperative. (**B**) XperCT volume axial postoperative. (**C**) XperCT volume coronal preoperative. (**D**) XperCT volume coronal post-operative.

In this case, we overlaid two XperCT volumes (pre-operative and post-operative) to assess the evolution of hydrocephalus (Figure 5). We noticed that there is a difference of 2–3 mm between the size of the ventricles at the beginning and at the end of the intervention for the embolization of the aneurysm of the anterior communicating artery, a difference that appeared in 90 min.

The overlying was performed automatically and quickly, as we were dealing with volumes with numerous bone landmarks. No intraoperative bleeding was detected, either, and the hyperdense appearance at the end of the intervention was due to the contrast media.

### 3.4. Case 4

**Figure 6:**
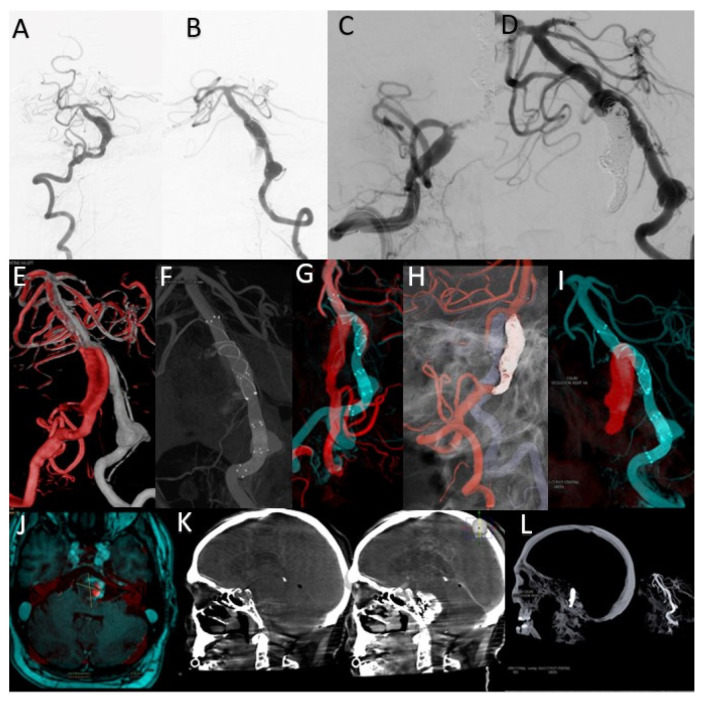
(**A**) Right VA preoperative DSA. (**B**) Left VA preoperative DSA. (**C**) Right VA postoperative DSA. (**D**) Left VA postoperative DSA. (**E**) Overlay between the right VA 3DRA (red) and left VA 3DRA (grey). (**F**) Left VA VasoCT MIP after stents deployment. (**G**) MIP Overlay between right VA 3DRA (red) and right VA VasoCT (blue) after stents deployment. (**H**) 3D Roadmap overlay between right VA 3DRA (red) and left VA 3DRA (grey) during the right VA distal segment occlusion. (**I**) MIP Overlay between the right VA coiled segment 3DRA (red) and right VA VasoCT (blue). (**J**) Overlay between the 1-month control MRI and left VA VasoCT at the end of the treatment. (**K**) Overlay between the XperCT preoperative sagittal volume and the XperCT postoperative sagittal volume. (**L**) Overlay between the XperCT postoperative sagittal volume and the pre-operative left VA 3DRA volume.

This case concerns a 43-year-old patient who presented to the emergency room for dysarthria and gait disturbances. He was diagnosed after imaging investigations with a fusiform aneurysm of the vertebrobasilar junction (Figure 6A,B).

The overlay between the two 3DRA volumes obtained by injection into the left and right vertebral arteries helped determine the best therapeutic strategy as stenting one vascular axis and sacrificing the other axis (Figure 6E). After analyzing the aneurysmal morphology and the vessels involved, it was decided to stent the left vertebrobasilar axis and occlude the final portion of the right vertebral artery.

By comparative analysis of the normal anatomy, we notice that the left PICA artery has its origin in the inferior portion of the aneurysmal sac while the right PICA artery has its origin at a distance from this aneurysm. Given these elements, the decision was made to preserve the right PICA by segmental closure of the vertebral artery between the stents placed on the right vertebrobasilar axis and the emergence of the artery (Figure 6C,D,I).

Moreover, the overlay of the 3DRA volume of the left vertebrobasilar axis with the VasoCT examination of the same axis, performed after telescopic placement of three stents (Figure 6F,G), helped to appreciate the areas of overlay between the distal and middle stent and also between the middle and proximal stent. As during MRI follow-up, a dimensional increase was observed (Figure 6J), and it was decided to place a new flow-diverter stent inside the first stent, with the purpose of covering the aneurysmal neck.

The sagittal XperCT volume pre-operative displayed near the sagittal XperCT volume post-operative, overlayed, but in mirror (independent function of Overlay XtraVision software), as in Figure 6K, provides information regarding potential bleeding from the giant basilar aneurysm in the posterior fossa, and can assess the mass effect and its relation with the posterior fossa craniectomy in such complex cases.

The analysis of these overlaying volumes is very useful as a result of the need for the subsequent placement of a FlowDiverter stent to cover the area of the dysplastic aneurysmal neck at the vertebrobasilar junction, as the segment with the active thrombus continues to recruit despite the initial placement of platinum coils inside the aneurysmal sac.

### 3.5. Case 5

A 65-year-old patient diagnosed with a Fisher IV subarachnoid hemorrhage in the emergency department. An AngioCT examination was performed, which revealed the presence of two cerebral aneurysms—one arising from the left PICA and the other from the left PCom, both of which the multidisciplinary medical team decided to treat in the same embolization session.

**Figure 7:**
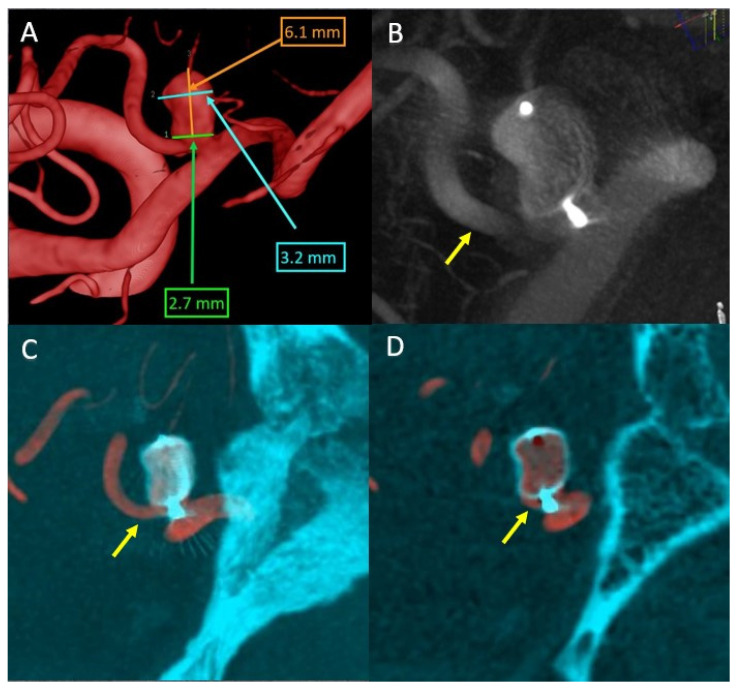
(**A**) Left posterior inferior cerebellar artery (PICA) aneurysm size—left vertebral artery 3DRA volume. (**B**) VasoCT Maximum Intensity Projection (MIP) WEB SL 7 × 3 mm. (**C**) MIP Overlay between the 3DRA volume from the diagnostic phase (red color) and the VasoCT (blue color) examination performed after WEB device detachment. (**D**) Thin-slice image reconstructions overlay between the 3DRA volume from the diagnostic phase (red color) and the VasoCT (blue color). Yellow arrow—left PICA.

Further, we will focus on the left PICA aneurysm, which was 3.2 × 2.7 mm in diameter, 6.1 mm in height, and 2.7 mm at the neck (Figure 7A). Since it has been shown that WEB devices are more efficient over time if an oversizing [13] is performed, after doing the measurements and a device simulation, a 7 × 3 mm device was selected. Given the need to preserve the left PICA and because it was decided to oversize by approximately 1 mm, to prove the good apposition of the device was crucial. This was difficult and only acheived with the help of a post-detachment VasoCT examination, as the device marker artifacts are projected over the parent artery (Figure 7B).

Thus, it was necessary to overlay the 3DRA volume during the diagnostic phase with the VasoCT examination performed after the device detachment (Figure 7C,D). 

In conclusion, the superposition shows good apposition in the aneurysmal sac and lack of protrusion at the level of the parent artery, with only the WEB device marker being projected at the level of the artery (and, therefore, no thromboembolic risk).

### 3.6. Case 6

**Figure 8:**
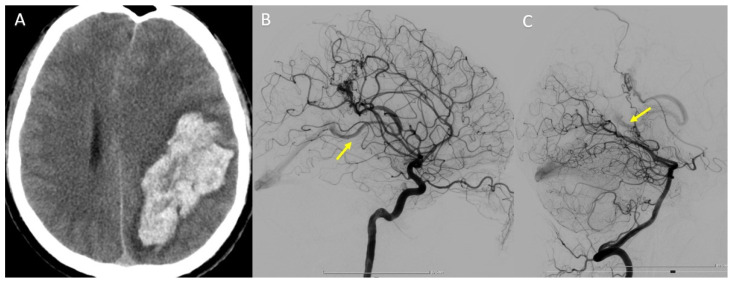
(**A**) Head CT scan—temporoparietal parenchymal hemorrhage. (**B**) Left internal carotid (ICA) DSA—lateral view. (**C**) Left vertebral artery (VA) DSA—lateral view. Yellow arrow—early filling of the deep venous system, and drainage to the great cerebral vein (of Galen).

In this case, we present a 34-year-old patient who was diagnosed with temporoparietal parenchymal hemorrhage following a ruptured cerebral AVM (Figure 8A). DSA revealed that the nidus is supplied from two distinct vascular territories (Figure 8B,C). 

**Figure 9:**
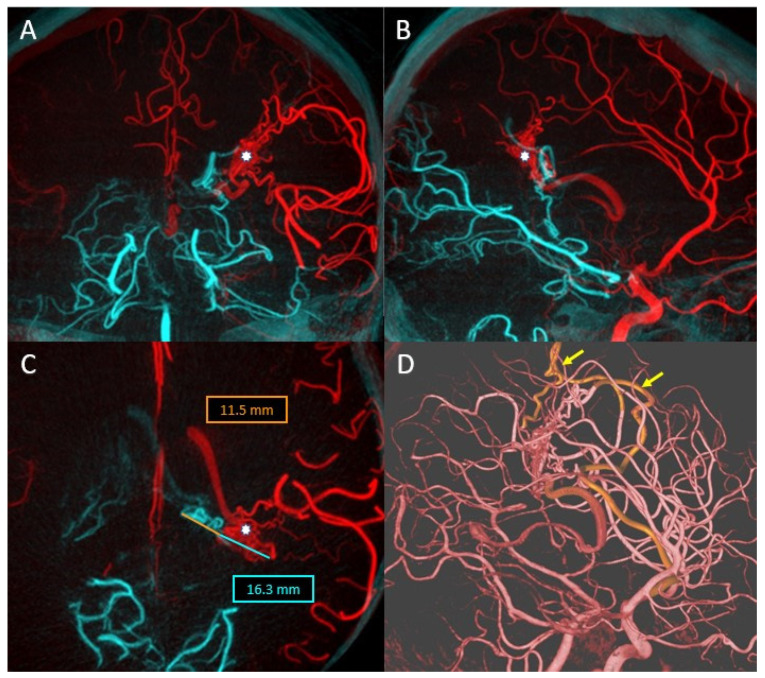
(**A**) Coronal MIP Overlay between the left ICA 3DRA (red) and left VA (blue). (**B**) Sagittal MIP Overlay between the left ICA 3DRA (red) and left VA (blue). (**C**) Axial MIP Overlay between the left ICA 3DRA (red) and left VA (blue)—nidus measurements. (**D**) MIP Overlay between the left ICA 3DRA and left VA—feeders targeted for endovascular occlusion. White asterisk—AVM nidus. Yellow arrow—AVM feeders targeted.

In order to choose the best treatment strategy, a detailed analysis of the 3DRA images as well as the overlay between the two 3D volumes obtained by injecting both the left internal carotid artery and the left vertebral artery was required (Figure 9A–C). The analysis of the images showed that the arterial feeders originated from the left MCA and left PCA, while the deep venous drainage merges into the vein of Galen. The 3DRA imaging demonstrates the existence of multiple intranidal aneurysms, the largest being located near the feeder from the MCA.

Considering the anatomy of the case, the treatment by catheterization of the branches going most directly towards the intranidal aneurysms (discovered during the diagnostic phase) was decided (Figure 9D). Thus, the analysis of the overlay between the two 3DRA volumes helped in the choice of an embolization strategy that allowed the exclusion of the AVM from the circulation by initial embolization of the nidal compartment at risk and secondary embolization of the remaining nidal compartment. 

By using this overlaying software, the risks associated with the intervention were greatly reduced, and by accurately choosing the target vessels from which to embolize, the operating times, and therefore the radiation dose, were greatly optimized.

## 4. Discussion

Pre-treatment analysis involves the superimposition of two 3DRA volumes of different vascular axes (left internal carotid artery and right internal carotid artery or left vertebral artery and right vertebral artery) with the amendment that both volumes must be acquired with the same FOV for the xtraVision station to allow their superimposition.

Regarding anterior communicating artery aneurysms with both the A1 segments patent, the fusion function of two 3D volumes of the left and right carotid axes yields determinant information, such as which of the two A1 segments has the larger diameter, i.e., the length of this segment in order to place a contralateral A1–A2 stent [14]. In some situations, Pagiola et al. observed asymmetry of the anterior communicating complex (about 80%) with the possibility of fenestrations or duplications of the anterior communicating artery [14].

In the case of cerebral aneurysms treated with either intrasaccular devices or stents, regardless of location, the information provided by this superimposition is useful in detecting the good apposition of devices in the intra-arterial lumen or aneurysm sac, the protrusion grade of a stent if placed in or near a bifurcation, or the degree of protrusion in the vascular lumen of an intrasaccular device. In order to respect the vascular regional anatomy, the operator who performs the overlay has to manually adjust the volumes in order to have a perfect superposition of the aneurysmal sac or the device itself present in two different acquisitions. By correlating this information with 2D imaging, therapeutic decisions can be guided towards either thrombolytic medication (if intraluminal thrombi are found) or choosing a new device that will correct the malposition of the first device (stent retubation).

Concerning vertebrobasilar junction aneurysms, usually fusiform, the involvement of both vertebral arteries makes essential the therapeutic decision to reconstruct a vertebrobasilar axis (left or right) and to occlude the contralateral vertebral axis. An important aspect is understanding the origins of the PICA arteries, the existence of a single PICA artery as well as the distance between its origin and the vertebrobasilar junction itself.

More complex treatments, such as using several stents in a telescopic manner or Y-stenting, mean that the individual analysis of the VasoCT volume as well as the overlap between the VasoCT and the initial 3DRA gives certainty of the correct placement of the stents used, and are an indispensable step in order to continue the treatment by placing coils and occluding the aneurysmal sac.

In cerebral AVMs endovascular treatement, the superimposition of the pre-embolization arterial 3DRA volume and the post-embolization XperCT volume accurately identifies the percentage of embolic material (Onyx, Squid, Phill) in the initial nidus as well as in the remaining compartment—circulating in the case of large AVMs and requiring multiple embolization sessions.

Using the overlay system, we are able to analyze each type of feeding artery supplying the AVM as well as the arterial supply to the normal brain, so we could find the best endovascular treatment strategy.

## 5. Limitations

The limitations of this study are its retrospective design, the non-randomized nature, and the small number of patients. Other limitations consist of the lack of balance among the aneurysmal location in which we used this type of imaging. We need large randomized prospective clinical studies to assess how AI can help in overlaying volume imaging sets during neurointerventional procedures. Superposed volumes in certain conditions provides, as seen (evaluation of the WEB device placement), better details than side-by-side (mirror) comparison [15]. 

## 6. Conclusions

The automatic overlay function based on artificial intelligence algorithms can reduce operator time by processing images quickly. The use of multiple sets of overlaying images is useful in treatment planning and for confirming the optimal apposition of the endovascular devices used. However, with current versions, it is often necessary to manually calibrate the images resulting from automatic processing. 

There is a need for further development of artificial intelligence-based technology to decrease the operating times and therefore the radiation doses, but also to increase the quality and safety of endovascular procedures. With the development of new artificial intelligence software and robotic technology in interventional neuroradiology, the addressability of procedures will increase. 

## Data Availability

Data available on request.
